# Unravelling the Role of PAX2 Mutation in Human Focal Segmental Glomerulosclerosis

**DOI:** 10.3390/biomedicines9121808

**Published:** 2021-12-01

**Authors:** Lorena Longaretti, Piera Trionfini, Valerio Brizi, Christodoulos Xinaris, Caterina Mele, Matteo Breno, Elena Romano, Roberta Giampietro, Giuseppe Remuzzi, Ariela Benigni, Susanna Tomasoni

**Affiliations:** Istituto di Ricerche Farmacologiche Mario Negri IRCCS, 24126 Bergamo, Italy; lorena.longaretti@marionegri.it (L.L.); piera.trionfini@marionegri.it (P.T.); valeriobrizi@yahoo.it (V.B.); christodoulos.xinaris@marionegri.it (C.X.); caterina.mele@marionegri.it (C.M.); matteo.breno@marionegri.it (M.B.); elena.romano@marionegri.it (E.R.); roberta.giampietro@marionegri.it (R.G.); giuseppe.remuzzi@marionegri.it (G.R.); ariela.benigni@marionegri.it (A.B.)

**Keywords:** PAX2, focal segmental glomerulosclerosis, induced pluripotent stem cells, podocytes, ureteric bud

## Abstract

No effective treatments are available for familial steroid-resistant Focal Segmental Glomerulosclerosis (FSGS), characterized by proteinuria due to ultrastructural abnormalities in glomerular podocytes. Here, we studied a private PAX2 mutation identified in a patient who developed FSGS in adulthood. By generating adult podocytes using patient-specific induced pluripotent stem cells (iPSC), we developed an in vitro model to dissect the role of this mutation in the onset of FSGS. Despite the PAX2 mutation, patient iPSC properly differentiated into podocytes that exhibited a normal structure and function when compared to control podocytes. However, when exposed to an environmental trigger, patient podocytes were less viable and more susceptible to cell injury. Fixing the mutation improved their phenotype and functionality. Using a branching morphogenesis assay, we documented developmental defects in patient-derived ureteric bud-like tubules that were totally rescued by fixing the mutation. These data strongly support the hypothesis that the PAX2 mutation has a dual effect, first in renal organogenesis, which could account for a suboptimal nephron number at birth, and second in adult podocytes, which are more susceptible to cell death caused by environmental triggers. These abnormalities might translate into the development of proteinuria in vivo, with a progressive decline in renal function, leading to FSGS.

## 1. Introduction

Familial steroid-resistant nephrotic syndrome (SRNS) is a subgroup of nephrotic syndromes classified by genetic mapping studies that progress to end stage renal disease (ESRD) [1]. Focal Segmental Glomerulosclerosis, the most important—but not the only renal lesion in SRNS—is characterized by the presence of partial sclerosis of some but not all glomeruli. Dysfunction of the glomerular podocyte, a specialized cell that forms the glomerular filtration barrier, is pivotal in the paradigm for the pathogenesis of FSGS [2]. Podocyte foot-process effacement is the main ultrastructural abnormality in FSGS and correlates with nephrotic-range proteinuria [3]. The discovery of mutations in various genes encoding podocyte proteins is one of the most exciting advances in the understanding of the pathogenesis of SRNS and has been made possible by studying familial forms [4]. The FSGS-associated genes identified so far encode proteins located in the podocyte slit diaphragm, cell membrane, cytosol, actin cytoskeleton, nucleus, mitochondria and lysosomes. By using exome sequencing, a disease-segregating missense heterozygous mutation (c.565G > A, p.G189R) in the *PAX2* gene has recently been identified in a large family with adult onset autosomal dominant FSGS [5]. We recently generated induced pluripotent stem cells (iPSC) from the proband of this family and fixed the *PAX2* c.565G > A point mutation using CRISPR/Cas9 technology, thus generating the proper isogenic control cell line [6]. Through this in vitro disease model, we were able to demonstrate that the *PAX2* c.565G > A point mutation was responsible for altered motility of the podocytes, which was restored after the gene editing process. These results strongly supported the hypo-thesis that PAX2 plays a role in the adult onset of FSGS. Moreover, since the PAX2 transcription factor regulates key developmental processes in kidney organogenesis [7,8], we investigated the role of the *PAX2* c.565G > A point mutation, both in affecting podocyte development and maturation and in ureteric bud morphogenesis.

## 2. Materials and Methods

### 2.1. Patient and Healthy Control

The patient and healthy control provided written informed consent for this study, which was approved by the Ethics Committee of Bergamo.

### 2.2. Human iPSC-Derived Podocytes Culture and Treatments

Control and patient-derived iPSC were differentiated into podocytes according to the protocol we developed previously [9]. From day 13 to day 18, iPSC-derived podocytes were cultured in RPMI 1640 medium (Gibco, Waltham, MA, USA) supplemented with 10% fetal bovine serum (FBS), ITS 1X (insulin–transferrin–selenium; ITS-G, 100X; Gibco), and 50 U/mL penicillin plus 0.05 mg/mL streptomycin (Gibco).

To evaluate cytoskeleton rearrangement, iPSC-derived podocytes at day 13 of diffe-rentiation were exposed to angiotensin II (Ang II, 500 nM, Sigma-Aldrich, St. Louis, MO, USA) for 24 h in DMEM-F12 + GlutaMAX (Gibco) in serum-free conditions.

To analyze susceptibility to apoptosis, at day 13 iPSC-derived podocytes were exposed to the puromycin aminonucleoside (PAN, 100 μg/mL, Sigma-Aldrich) in DMEM-F12 + GlutaMAX supplemented with 1% FBS.

### 2.3. Gene Expression Analysis

Total RNA was isolated with Trizol Reagent (Invitrogen, Waltham, MA, USA) and treated with DNAse (Promega, Madison, WI, USA), as described by the manufacturer. Total RNA (2 μg) was used for reverse transcription reaction with the SuperScript VILO cDNA Synthesis kit (Invitrogen, Waltham, MA, USA), following the manufacturer’s instructions. Quantitative real-time PCR assays were performed using SYBR Green PCR Master Mix with primers or Taqman PCR Master Mix with predesigned Taqman probes for the genes of interest according to the supplier’s recommendations (Applied Biosystems, Waltham, MA, USA) (Table 1). Gene expression levels were normalized to the housekeeping gene HPRT1.

### 2.4. Immunofluorescence Analysis

Cells were fixed with phosphate-buffered saline (PBS) containing 4% paraformaldehyde (Società Italiana Chimici, Rome, Italy) for 15 min at room temperature (RT). After three washes with PBS, the cells were permeabilized with 0.3% Triton X-100 (Sigma-Aldrich) for 10 min at RT (when necessary) and then incubated with 5% bovine serum albumin (BSA, Sigma-Aldrich) for 1 h as a blocking solution. The samples were stained with primary antibodies diluted in 2% BSA solution overnight at 4 °C, followed by the corresponding Alexa 546- or Alexa 488-conjugated secondary antibodies for 1 h at RT. F-actin filaments were stained with TRITC-conjugated phalloidin (Invitrogen). Nuclei were counterstained with 4′,6-diamidino-2-phenylindole (DAPI, Sigma-Aldrich). Images were taken using an inverted confocal laser-scanning microscope (LS 510 Meta) or the Axio Observer Z1 fluorescence microscope (Carl Zeiss, Oberkochen, Germany). The primary antibodies used are listed in Table 2.

### 2.5. Genome-Wide Differential Gene Expression Analysis

Two replicates for each combination of genotype and time (control and patient iPSC-derived cells, at days 6, 13 and 18) were used for the genome-wide differential gene expression analysis. Total RNA was extracted using the PureLink RNA Micro Scale Kit (Invitrogen) and cDNA libraries were prepared according to the Ion Ampliseq Transcriptome Human Gene Expression Kit (Ion Torrent, Guilford, CT, USA) protocol, using ten nanograms of high-quality RNA from each sample. Briefly, cDNA was first generated with the SuperScript VILO cDNA Synthesis kit. Then, cDNA was amplified using the Ion AmpliSeq technology to accurately maintain expression levels of all targeted genes. After amplification, the resulting amplicons were ligated to barcode adapters. Libraries were then quantified by qPCR using the Ion Library Quantitation Kit (Ion Torrent) and pooled in equimolar amounts. Template preparation (emulsion PCR) and chip loading were performed on an Ion Chef instrument (Ion Torrent). Each library was sequenced twice for a total of three runs of the Ion 540 chip. Libraries were sequenced on the Ion S5XL Sequencer (Ion Torrent). Gene expression levels were provided as raw read counts. As the Ion AmpliSeq Transcriptome Human Gene Expression Kit covers each target gene with a single amplicon, normalization for transcript length bias was unnecessary. To compare gene expression levels between patient and control iPSC-derived nephron progenitors or podocytes, we performed differentially expressed gene (DEG) analysis on raw read counts u-sing DESeq2 [10], after discarding targets with less than 10 reads in all samples. DEGs, selected based on their adjusted p value and/or log2 fold change, were submitted to enrichment analysis using Enrichr [11]. Read counts were normalized by the regularized logarithm (rlog) method implemented in DESeq2 before drawing heat-maps. Correction for multiple tests was carried out with the Benjamini–Hochberg procedure.

### 2.6. Albumin Uptake Assay

An albumin uptake assay was performed as previously described [12]. Human iPSC-derived podocytes at 13 days of differentiation were incubated with serum-free medium overnight. After washing with Ringer’s buffer pH 7.4, cells were exposed to 1 mg/mL FITC-conjugated BSA for 90 min at 37 °C. After washing with Ringer’s buffer, cells were fixed in 4% PFA for 10 min at room temperature. Nuclei were stained with DAPI. Cells were mounted using Dako Fluorescence Mounting Medium (DAKO, Glostrup, Denmark) and examined by fluorescence microscope (ApoTome Axio Imager Z2, Zeiss). The percentage of albumin-positive cells was defined as the percentage of FITC-positive cells/DAPI-positive cells per field.

### 2.7. Cell Viability Assay

Cell viability was assessed using a 3-(4,5-dimethylthiazol-2-yl)-5-(3-carboxymethoxyphenyl)-2-(4-sulfophenyl)-2H-tetrazolium (MTS) colorimetric assay (CellTiter96^®^Aqueous One Solution Cell Proliferation, Promega) according to the ma-nufacturer’s instructions. Human iPSC-derived podocytes were seeded in 96-well culture plates at a density of 5.5 × 10^3^ cells per well in DMEM-F12 + GlutaMAX medium with 1% of FBS and allowed to adhere. After 12 h the supernatant was discarded and the cells were exposed to PAN (100 μg/mL). After 24 h of treatment, MTS tetrazolium compound (final concentration of 0.5 mg/mL) was added for 1.5 h at 37 °C. The amount of soluble formazan produced by a reduction of MTS by viable cells was determined by measuring the absor-bance at 490 nm using a micro-plate reader (Infinite m200-pro, Tecan Group Ltd., Männedorf, Switzerland). Background 490 nm absorbance given by medium alone was subtracted from the average absorbance of cultured cells.

### 2.8. Enzyme-Linked Immunosorbent Assay

The measurement of IGF1 protein in the cell supernatant was performed by using a commercially available enzyme-linked immunosorbent assay kit (R&D Systems) follo-wing the manufacturer’s instructions. The minimum detectable dose of human IGF1 ranged from 0.004–0.022 ng/mL. No significant cross-reactivity or interference was observed with other IGF isoforms.

### 2.9. Differentiation of iPSCs toward Ureteric Bud (UB) Progenitor-like Cells and Tubule Engineering

Human iPSC were induced to differentiate toward UB-like cells as previously described [13]. Briefly, cells were grown for 2 days in a chemically defined basal medium [DMEM/F12 + GlutaMAX, 17.5 mg/mL BSA fraction V (Merck Millipore, Burlington, MA, USA), 17.5 μg/mL human insulin (Sigma-Aldrich), 275 μg/mL human holo-transferrin (Sigma-Aldrich), 450 μM 1-thioglycerol (Sigma-Aldrich), 0.1 mM non-essential amino a-cids (Gibco), 1% Pen-Strep] supplemented with 50 ng/mL human fibroblast growth factor 2 (FGF2) (Peprotech, Rocky Hill, CT, USA) and 30 ng/mL human bone morphogenetic protein 4 (BMP4) (Peprotech). For the next 2 days, cells were exposed to basal medium supplemented with 1 μM all-trans retinoic acid (Sigma-Aldrich), 10 ng/mL human Activin A (Peprotech) and 100 ng/mL human bone morphogenetic protein 2 (BMP2) (Abnova, Heidelberg, Germany).

For tubule engineering, differentiated iPSC were harvested using Accutase (Gibco), centrifuged at 300× *g* for 3 min, resuspended in 2.4 mg/mL rat tail collagen type I (Corning, Corning, NY, USA) on ice, and seeded in the PDSM macro-scaffolds at a concentration of 1.2 × 10^5^ cells/μL collagen. After collagen polymerization, medium was added on the scaffold surface and tubules were cultured in static conditions for up to 2 days in basal medium supplemented with 1% FBS, 40 ng/mL HGF and 100 ng/mL glial cell-derived neurotrophic factor (GDNF) (Abcam, Cambridge, UK). Cultures were monitored using light microscopy for up to 2 days without changing the medium. The overall budding events were quantified in bright-field images of UB-like tubules. Data are expressed as percentage of ramified buds over total buds that emerged from human tubules.

### 2.10. Statistical Analysis

Statistical analysis was performed using GraphPad Prism software (version 9). Data were analyzed using ANOVA followed by the Bonferroni test for multiple comparisons or *t* test for unpaired data, as appropriate. Data were presented as mean values ± standard deviation (SD). Statistical significance was defined as *p* < 0.05.

## 3. Results

### 3.1. Generation and Characterization of PAX2^G189R/+^ iPSC-Derived Podocytes

**Figure 1:**
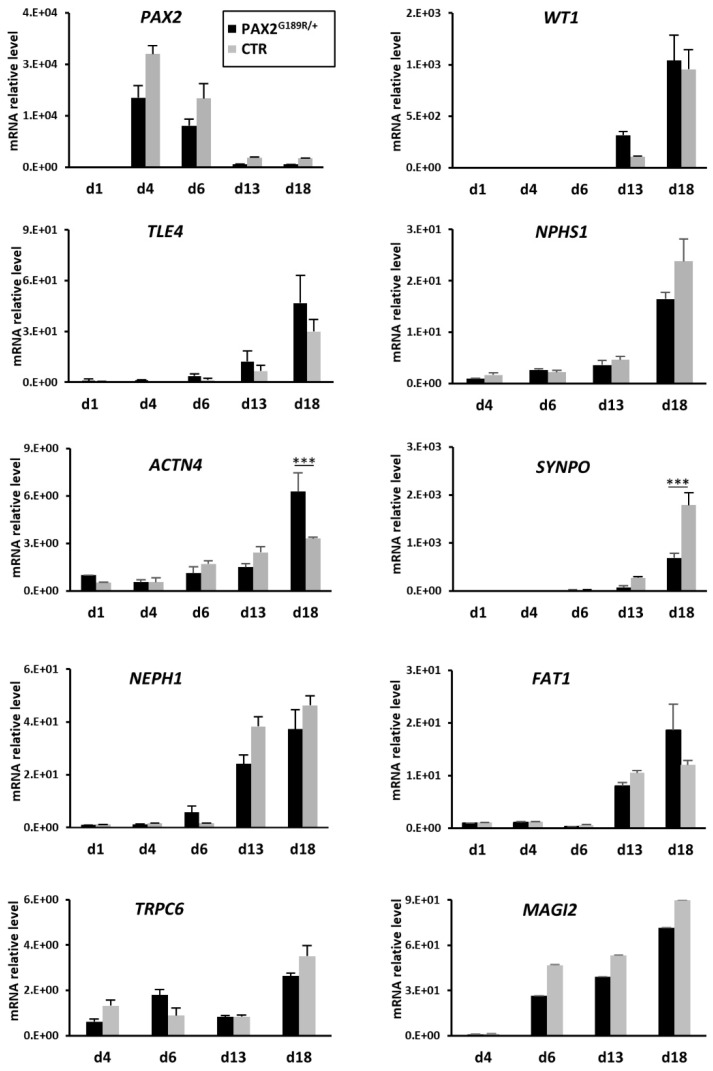
Temporal expression levels of stage-specific marker genes at the indicated time points (*n* = 3) in cells derived from CTR and PAX2^G189R/+^ iPSC and differentiated towards podocytes. Data are expressed as mean ± SD (*** *p* < 0.0001).

**Figure 2:**
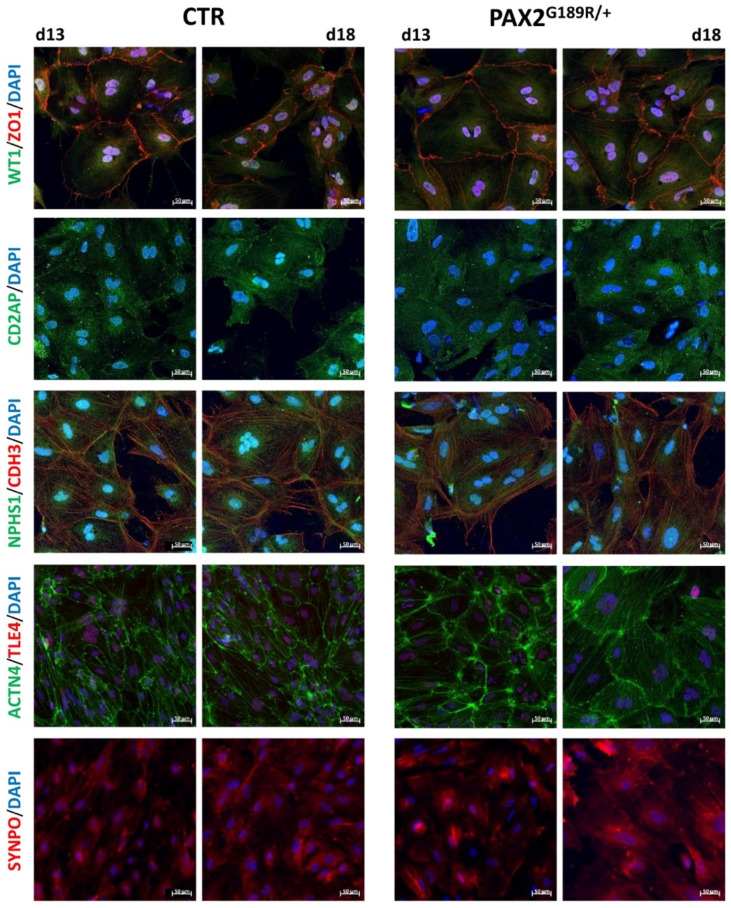
Representative images of specific markers in podocytes derived from CTR and PAX2^G189R/+^ iPSC (*n* = 4).

Using a stepwise protocol that we developed previously [9], we differentiated control and PAX2^G189R/+^ iPSC into podocytes. In a set of experiments, podocytes were also maintained from day 13 to day 18 in a medium specific to the human podocyte cell line [14]. Real time PCR analysis revealed that *PAX2* peaked in the first stage of differentiation, confirming the proper induction of intermediate mesoderm-like cells (Figure 1). At day 6 of the differentiation protocol, *SIX2*, a marker that characterizes a population of self-renewing multipotent nephron progenitors (NP), was half expressed in PAX2^G189R/+^ NP than in control cells without compromising cell differentiation (Supplementary Figure S1). Indeed, all the podocyte markers were strongly induced soon after 13 days of differentiation to a similar extent in control and patient podocytes, as confirmed by gene expression ana-lysis (Figure 1). Although *SYNPO* and *ACTN4* appeared to be significantly modulated between the control and the patient at day 18, no major differences in the expression of the corresponding proteins were observed between control and patient podocytes either at day 13 or day 18 of differentiation (Figure 2).

### 3.2. Functional Properties of PAX2^G189R/+^ iPSC-Derived Podocytes

**Figure 3:**
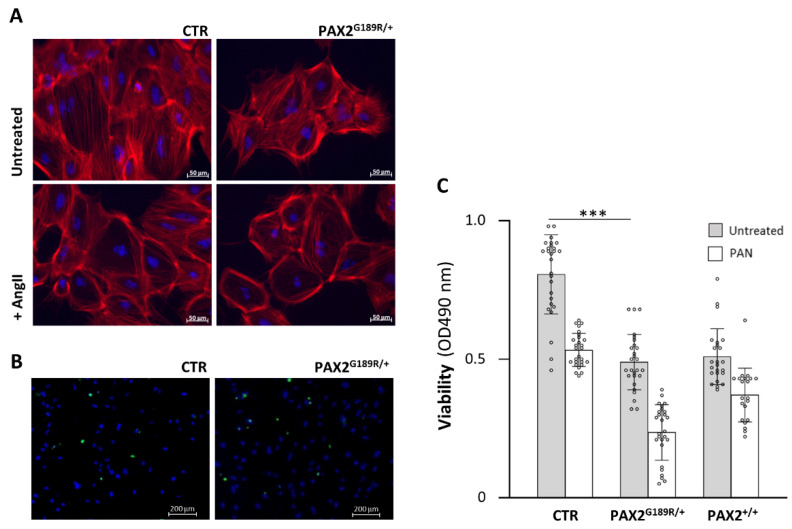
iPSC-derived podocytes functional assays at day 13 of differentiation. (**A**) Representative F-actin immunostaining of CTR and PAX2^G189R/+^ podocytes after 24 h of Ang II exposure (*n* = 3). (**B**) Assessment of endocytosis ability by FITC-albumin uptake of CTR and PAX2^G189R/+^ podocytes (*n* = 3). (**C**) Viability of CTR, PAX2^G189R/+^ and PAX2^+/+^ podocytes after 24 h exposure to PAN. Data are expressed as mean ± SD (*** *p* < 0.0001 untreated CTR versus untreated PAX2^G189R/+^ podocytes) and are derived from at least *n* = 21 measurements/sample from four independent experiments.

To evaluate the functional properties of patient podocytes, we exposed control and patient podocytes to Ang II, one of the major players in podocyte injury. As shown in Figure 3A, Ang II induced actin cytoskeleton rearrangement to a similar degree in control and patient podocytes (Figure 3A). Similarly, the ability to endocytose albumin was not compromised in patients’ cells (% of albumin-positive cells: 7.95 ± 3.04 in PAX2^G189R/+^ podocytes vs. 7.71 ± 3.62 in control podocytes, Figure 3B).

Based on the evidence that the patient developed nephrotic syndrome in adulthood, we hypothesized that a second hit was required to develop FSGS. Translating to our in vitro model, patient podocytes might have an impaired response to environmental triggers. To test this hypothesis, we treated podocytes with PAN, a podocytotoxin broadly used for modelling glomerular diseases in vitro and in experimental models due to its effect on inducing podocyte apoptosis, cytoskeleton damage and autophagy [15]. We performed an MTS assay to study podocyte viability. Patient podocytes were significantly less viable than control podocytes before damage induction (Figure 3C). PAN induced a more robust reduction in cell viability in patient versus control podocytes. Indeed, the percentage of apoptotic cells after PAN was 33% in CTR and 52% in patient cells (*p* = 0.0003), suggesting that patient cells were more susceptible to damage (Supplementary Figure S2). To evaluate whether the G189R-PAX2 mutation could account for this effect, we assessed cell viability in podocytes obtained from patient CRISPR/Cas9-edited iPSC, where the disease-causing mutation was the sole variable that had been modified. The correction of the mutation protected edited podocytes from PAN-induced damage. Indeed, the percentage of apoptotic cells was restored to levels comparable to those of control cells (Supplementary Figure S2). In contrast, editing the *PAX2* mutation did not affect podocyte viability before damage induction, which remained at the same levels as in patient podocytes (Figure 3C), suggesting that the genetic background might play a role in determining cell viability.

### 3.3. Gene Expression Signature of Control and PAX2^G189R/+^ iPSC-Derived Podocytes

**Figure 4:**
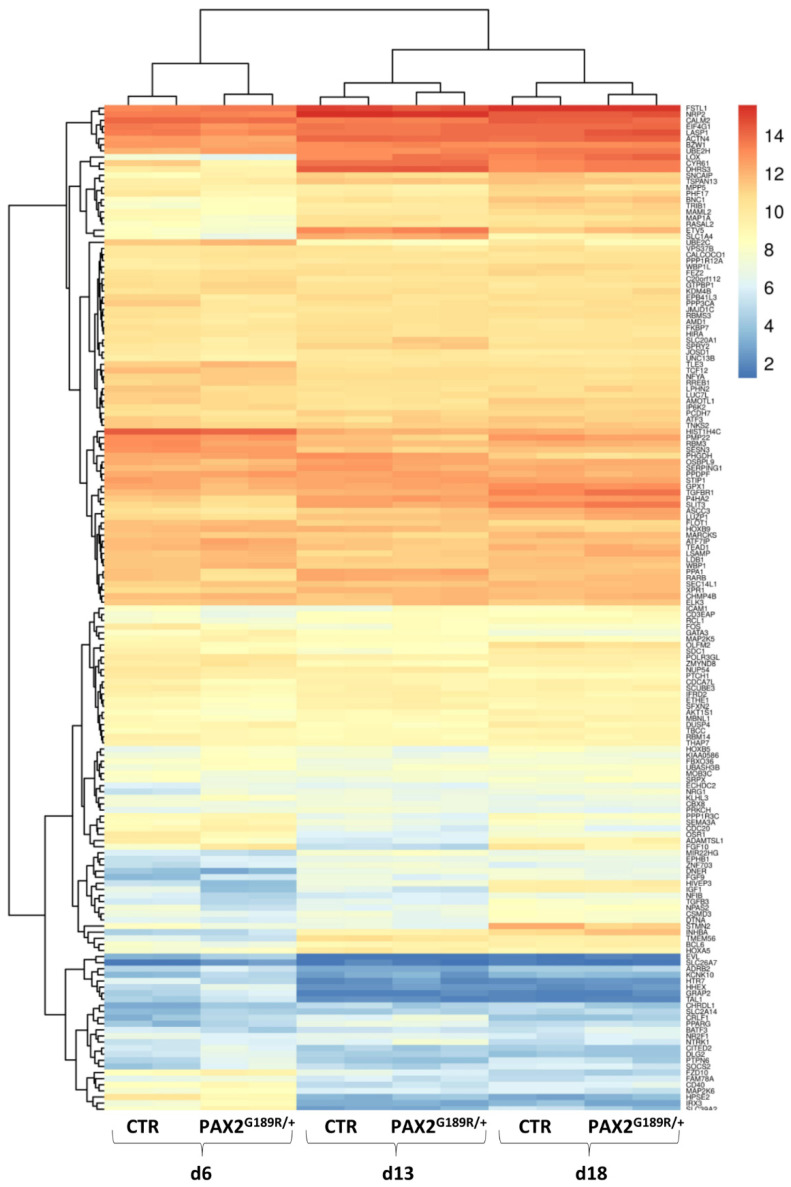
Whole-transcriptomic analysis of CTR and PAX2^G189R/+^ derived NP (day 6) and of CTR and PAX2^G189R/+^ derived podocytes at day 13 and 18 of differentiation: heatmap of log2 expression levels of the top 165 differentially expressed PAX2 target genes.

**Figure 5:**
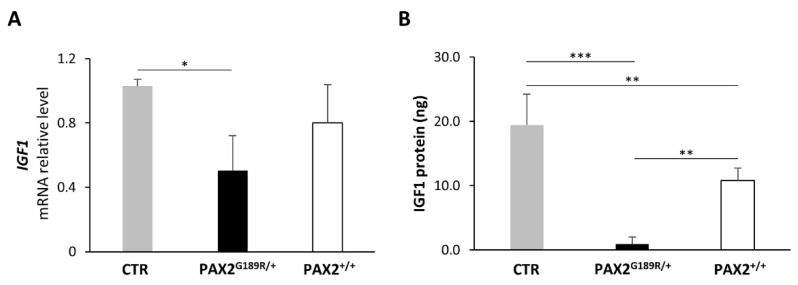
(**A**) Gene expression analysis of *IGF1* in CTR, PAX2^G189R/+^ and PAX2^+/+^ podocytes (*n* = 4). (**B**) Assessment of IGF1 release by ELISA in CTR, PAX2^G189R/+^ and PAX2^+/+^ podocytes (*n* = 4). All assays were performed in podocytes at day 13 of differentiation. Data are expressed as mean ± SD (* *p* < 0.05, ** *p* < 0.005, *** *p* < 0.0001).

Whole-transcriptomic analysis was performed on control and patient NP at day 6 of differentiation and on control and patient podocytes at 13 and 18 days of differentiation. The analysis did not reveal any major differences between control and patient cell signatures either in NP or podocytes at day 13 or day 18 of differentiation, even in PAX2 target genes (Figure 4). Notably, *IGF1*, one likely PAX2 target, was downregulated in patient podocytes at day 13 of the differentiation protocol. IGF1 is a soluble peptide that regulates podocyte survival [16]. We first validated the *IGF1* mRNA downregulation using real-time PCR (Figure 5A) and confirmed its downregulation at the protein level, as well (Figure 5B). Following the correction of the G189R-PAX2 mutation, IGF1 levels significantly increased without, however, reaching those of control cells (Figure 5A,B). This may be due to intrinsic differences between control and patient cells, or to additional, potentially dysregulated, mechanisms affecting IGF1 expression.

**Figure 6:**
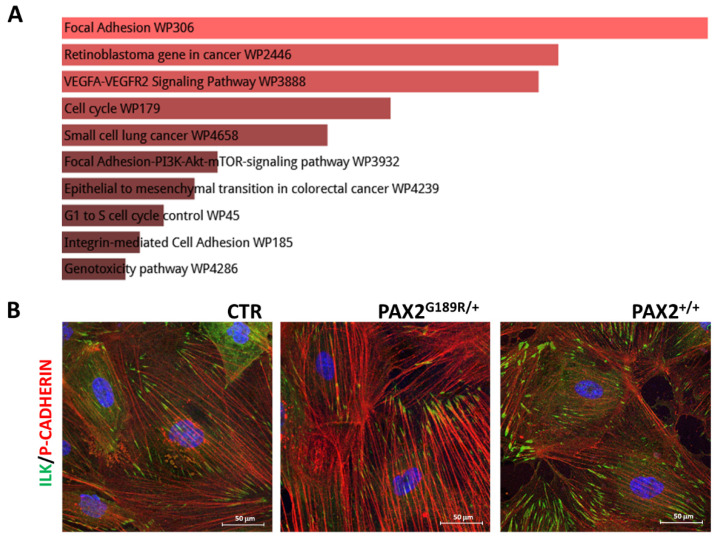
(**A**) Gene set enrichment analysis of the top 1000 differentially expressed genes between control and patient podocytes using Enrich WikiPathway 2021 Human. (**B**) Representative ILK/P-CADHERIN immunostaining in CTR, PAX2^G189R/+^ and PAX2^+/+^ derived podocytes at day 13 of differentiation (*n* = 3).

Enrichment analyses of the transcriptomic profile revealed that the focal adhesion pathway was one of the mostly dysregulated pathways in patient podocytes (Figure 6A). Focal adhesions are integrin-based multiprotein complexes that are tightly associated with the actin cytoskeleton and are essential for cell interaction with the glomerular basement membrane [17]. Defects in the focal adhesion pathway could explain why patient podocytes are more susceptible to cytotoxic molecules. Analysis of the integrin linked kinase (ILK), a focal adhesion protein, revealed altered spatial organization in patient podocytes, compared to control ones (Figure 6B). Notably, when we assessed ILK distribution in podocytes obtained from patient CRISPR/Cas9 edited iPSC, we observed that the correction of the mutation was able to rescue their phenotype.

### 3.4. Correction of G189R-PAX2 Restores Ureteric Bud Morphogenesis Potential

**Figure 7:**
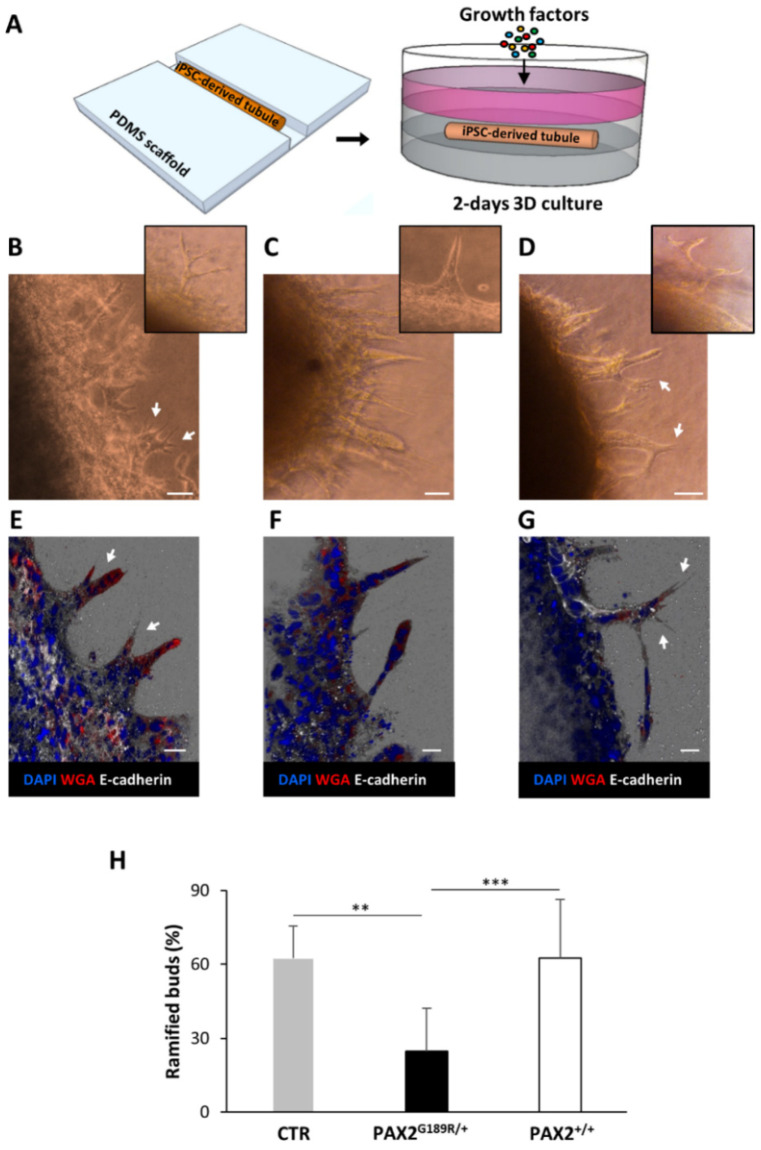
3D-engineered tubules for studying PAX2^G189R/+^ developmental defects. (**A**) Experimental design;bright field images and E-cadherin expression analysis of (**B**,**E**) control (*n* = 9), (**C**,**F**) patient (*n* = 13) and (**D**,**G**) editing-derived tubules (PAX2^+/+^, *n* = 12), respectively. (**H**) percentage of the ramified buds (arrows) in the total buds emerged in the different clones. Scale bars: (**B**–**D**): 50 µm; (**E**–**G**): 25 µm. Data are expressed as mean ± SD (** *p* = 0.0002; *** *p* < 0.0001) from three independent experiments.

PAX2 plays a key role in ureteric bud (UB) morphogenesis [7,18] and its heterozygous mutations cause serious developmental defects. To study the effect of the G189R-PAX2 mutation on UB development, we engineered UB-like tubules using control, mutant and edited cells, and compared their morphogenetic capacities, as previously described [13] (Figure 7). Quantification analysis revealed a marked reduction in ramified buds in patient-derived tubules compared to controls. Interestingly, the correction of the point mutation totally restored normal UB-like tubule budding, further confirming the deleterious effect of this mutation on kidney development and highlighting the therapeutic re-levance of gene editing in this patho-phenotype.

## 4. Discussion

Mutations in PAX2 were previously associated with rare congenital abnormalities of the kidney and urinary tract (CAKUT) and with renal coloboma syndrome (RCS), while little was known about the effects of PAX2 mutations in diseases affecting the glomerular components of the kidney. While most of the FSGS-associated PAX2 mutations have mainly been located in the N-terminal PAX2 region, leading to the inhibition of PAX2 binding to DNA, the patient we studied here carried a heterozygous mutation (G189R) affecting the PAX2 central octapeptide domain, a functionally important and conserved region. This mutation was predicted with high confidence to be damaging. To test this hypothesis, we developed a specific in vitro model by generating iPSC from patients’ PBMC and developed a protocol to differentiate iPSC into adult podocytes, a critical cellular element in maintaining renal function. The differentiation protocol mimics the developmental stages of the metanephric mesenchyme component of the kidney, from which renal tubules and glomeruli originate, making it possible to evaluate defects in the developmental process. We observed that during the developmental stages from intermediate mesoderm (IM) to NP the induction of *SIX2*, an NP marker that promotes IM-like cell maturation into renal progenitors, was significantly lower in NP derived from patient cells compared to control ones. However, the reduced *SIX2* expression did not compromise cell differentiation. Patient NP were able to properly differentiate into adult podocytes that shared the same markers of control podocytes and were similarly able to respond to stressors and to internalize albumin. Instead, patient iPSC-derived podocytes were less viable and more susceptible to cell injury when exposed to PAN than control podocytes. Increased susceptibility of patient cells to apoptotic damage could be the result of the abnormal distribution of focal adhesion complexes affecting podocyte anchoring to the glomerular basement membrane. Whole-transcriptomic analysis confirmed the central role of the dysregulated focal adhesion pathway in patient iPSC-derived podocytes. This could translate in vivo into an inability of podocyte to adequately counteract physiological hydrodynamic forces acting on the glomerular capillary wall as recently demonstrated in a rat experimental model of FSGS [19]. In an attempt to resist to the applied forces and prevent detachment, podocytes deposit additional GBM components, leading to a disorganized GBM, which finally leads to disease progression and kidney failure [20]. We also observed changes in the IGF1 expression in PAX2^G189R/+^ podocytes that could further explain why cells are more susceptible to apoptosis when exposed to PAN. Previous studies demonstrated that podocytes express IGF1 and its receptor IGF1R in control human glomeruli and that IGF1 protects podocytes from apoptosis in vitro [16,21] suggesting that an autocrine IGF1/IGF1R signaling may play a role in podocyte survival. Notably, dysregulation of the IGF1 system has been described in several kidney diseases, including diabetic nephropathy, polycystic kidneys and proteinuric kidney diseases [22,23]. According to our data, analysis of the glomerular transcriptomic dataset available on Nephroseq showed that *IGF1* was downregulated in focal segmental glomerulosclerosis patients compared to healthy controls (https://www.nephroseq.org/resource/main.html; accessed on 24 Sempember 2021).

All the above results are in line with the clinical history of the patient who developed nephrotic syndrome only in adulthood, following a viral infection, and underwent kidney transplantation 27 years later. In our previous study, we showed that patient podocytes exhibited reduced cell motility that was normalized when the mutation was fixed using the CRISPR/Cas9 technique [6]. Here, gene editing rescued cell susceptibility to PAN but not the cell viability of untreated cells. Moreover, the IGF1 production—although significantly rescued—did not reach the levels of the control. These data suggest that the genetic background, apart from *PAX2*, may play an additional role. Indeed, exome sequencing of the proband and of an affected family member identified 24 additional mutated genes in addition to *PAX2*, that ranked first following a prioritization analysis after functional annotation and given its known role in CAKUT and RCS [5]. Correction of the mutation restored the distribution of cytoskeletal proteins that play a fundamental role in podocyte adhesion to the glomerular basal membrane [24,25] and strengthen our previous data on the altered motility of the patient podocytes.

It is known that PAX2 heterozygous mutations can lead to a reduction in ureteric bud branching and nephron numbers [26]. To test the impact of the G189R mutation, we used a method that mimicked the early developmental process of ureteric bud branching and allowed for the quantification of morphogenetic events. The mutation found in our patient significantly impaired ureteric bud developmental capacity, which was restored in edited cells. This developmental defect could be responsible for a reduced number of nephrons at birth, which, together with the increased susceptibility of adult podocytes to injury, may account for the adult onset of FSGS.

These results are a step forward in understanding nephrotic syndrome and specifically in gaining knowledge of the pathogenic mechanisms underlying FSGS associated with PAX2 mutations, which account for 4% of adult FSGS. Our findings support the hypothesis that the G189R-PAX2 mutation has a dual effect in inducing the adult onset of FSGS: one, the most relevant for the onset of the pathology, in the early stage of kidney development, that by compromising ureteric bud development may predispose the kidney to the development of FSGS, and a second effect in adult podocytes, which are not able to properly counteract environmental triggers, making them more susceptible to injury. Further studies will be important for better defining the role of additional mutated genes found in patient cells in the development of FSGS.

## Figures and Tables

**Table 1:** List of Taqman probes and primers.

**Catalog Number**	**Gene Symbol**	**Description**
Hs99999909_m1	*HPRT1*	Hypoxanthine phosphoribosyltransferase 1
Hs01057416_m1	*PAX2*	Paired box 2
Hs00240913_m1	*WT1*	Wilms tumor 1
Hs00702468_s1	*SYNPO*	Synaptopodin
Hs00989190_m1	*TRPC6*	Transient receptor potential cation channel subfamily C member 6
**RefSeq**	**Gene Symbol**	**Sequence 5′-3′**
NM_000194.2	Hs *HPRT1*	Fwd: GGCAGTATAATCCAAAGATGGTCA Rev: TCCTTTTCACCAGCAAGCTTG
NM_016932	Hs *SIX2*	Fwd: CTTGCCACCGTTCATTCT Rev: GGACCAGGACACAGAGTA
NM_0013515421	Hs *TLE4*	Fwd: CCATCATTGGGCAGCAACAAC Rev: CTACCGATGGGTGGAATGGC
NM_004646.3	Hs *NPHS1*	Fwd: GGCCACAGCCAGGGTGA Rev: ATGGGGGCCTCCAGTGC
NM_004924.4	Hs *ACTN4*	Fwd: GCCCGATCTCCTCCATCTTG Rev: CCCTGGATGAACTTAGAGCCC
NM_005245.3	Hs *FAT1*	Fwd: CCTCACGGTCATGGTACGAG Rev: AAACCCGCCCTTTGTAGGAG
NM_001286349.1	Hs *NEPH1*	Fwd: AGGTGCCGCTCTATGTGAAC Rev: TTCCAGGCCCATGCTATGC
NM_012301.3	Hs *MAGI2*	Fwd: AAGTAGGCAACAAGTGCCACC Rev: GCCAAATCCAGACTCCATCCT
NM_001111283.2	Hs *IGF1*	Fwd: TGTACTTCAGAAGCAATGGGAA Rev: TGGTGTGCATCTTCACCTTCA

**Table 2:** List of primary antibodies.

Antibody	Source	Catalog Number	Working Dilution
Alpha-Actinin 4	Origene	TA307264	1:200
CD2AP	Santa Cruz	SC-9137	1:300
CDH3	R&D System	MAB861	1:100
Integrin linked ILK	Abcam	ab76468	1:100
NPHS1	Santa Cruz	SC-28192	1:100
P-cadherin	R&D System	MAB861	1:50
Paxillin	Genetex	GTX125891	1:200
Synaptopodin	Abcam	ab101883	1:100
TLE4	Santa Cruz	SC-365406	1:150
WT1	R&D System	AF5729	1:50
ZO-1	Invitrogen	61-7300	1:100

## Data Availability

Transcriptomic data have been deposited in NCBI’s Gene Expression Omnibus (Edgar et al., 2002) and are accessible through GEO Series accession number GSE185558 (https://www.ncbi.nlm.nih.gov/geo/query/acc.cgi?acc=GSE185558; accessed on 30 November 2021).

## Supplementary Materials

The following are available online at https://www.mdpi.com/article/10.3390/biomedicines9121808/s1, Figure S1: Temporal expression levels of *SIX2* in control and patient cells; Figure S2: Evaluation of PAN induced injury.
