# Supplementary material for: Unravelling the Role of PAX2 Mutation in Human Focal Segmental Glomerulosclerosis
Source: Biomedicines. 2021 Dec 1;9(12):1808. doi: 10.3390/biomedicines9121808 (PMC8698597; doi:10.3390/biomedicines9121808)
Supplement: Supplementary file 1 [file biomedicines-09-01808-s001.zip › biomedicines-1459574-supplementary.pdf]

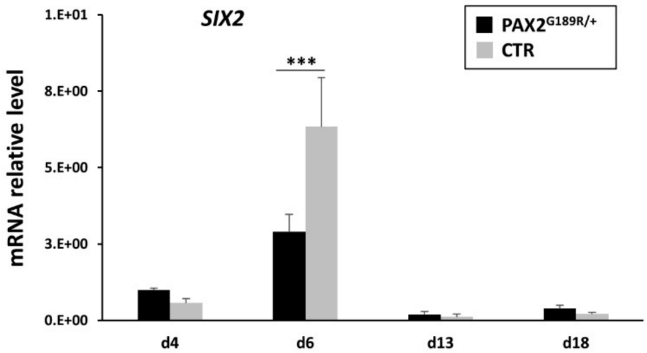

Figure S1. Temporal expression levels of *SIX2* in control and patient cells. Gene expression analysis of *SIX2* at the indicated time points (n = 4) in cells derived from CTR and PAX2$^{G189R/+}$ iPSC and differentiated towards podocytes. Data are expressed as mean ± SD (***p < 0.0001).

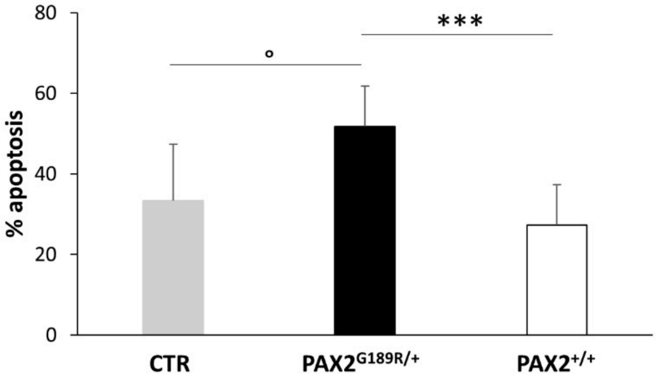

Figure S2. Evaluation of PAN-induced injury. Percentage of apoptosis of CTR, PAX2$^{G189R/+}$ and PAX2 $^{+/+}$ podocytes after 24h exposure to PAN. The percentage of apoptosis in each experimental group (CTR, PAX2$^{G189R/+}$ and PAX2 $^{+/+}$ podocytes) was calculated as difference of viability between untreated and PAN-treated cells, settling as 100% cell viability of untreated cells. Data are expressed as mean ± SD (°p = 0.0003; ***p < 0.0001).
